# In search for interplay between stool microRNAs, microbiota and short chain fatty acids in Crohn’s disease - a preliminary study

**DOI:** 10.1186/s12876-020-01444-3

**Published:** 2020-09-21

**Authors:** Filip Ambrozkiewicz, Jakub Karczmarski, Maria Kulecka, Agnieszka Paziewska, Magdalena Niemira, Natalia Zeber-Lubecka, Edyta Zagorowicz, Adam Kretowski, Jerzy Ostrowski

**Affiliations:** 1 Department of Genetics, Maria Sklodowska-Curie National Research Institute of Oncology, Roentgena 5, 02-781 Warsaw, Poland; 2 Department of Gastroenterology, Hepatology and Clinical Oncology, Centre of Postgraduate Medical Education, 02-781 Warsaw, Poland; 3 Clinical Research Centre, Medical University of Bialystok, Białystok, Poland; 4 Department of Oncological Gastroenterology, Maria Sklodowska-Curie National Research Institute of Oncology, 02-781 Warsaw, Poland

**Keywords:** Crohn’s disease, 16S rRNA, miRNA, SCFAs, Biomarker

## Abstract

**Background:**

Inflammatory bowel diseases are classic polygenic disorders, with genetic loads that reflect immunopathological processes in response to the intestinal microbiota. Herein we performed the multiomics analysis by combining the large scale surveys of gut bacterial community, stool microRNA (miRNA) and short chain fatty acid (SCFA) signatures to correlate their association with the activity of Crohn’s disease (CD).

**Methods:**

DNA, miRNA, and metabolites were extracted from stool samples of 15 CD patients, eight with active disease and seven in remission, and nine healthy individuals. Microbial, miRNA and SCFA profiles were assessed using datasets from 16S rRNA sequencing, Nanostring miRNA and GC-MS targeted analysis, respectively.

**Results:**

Pairwise comparisons showed that 9 and 23 taxa differed between controls and CD patients with active and inactive disease, respectively. Six taxa were common to both comparisons, whereas four taxa differed in CD patients. α-Diversity was lower in both CD groups than in controls. The levels of 13 miRNAs differed (*p*-value < 0.05; FC > 1.5) in CD patients and controls before FDR correction and 4 after. Of six SCFAs, the levels of two differed significantly (*p*-value < 0.05, FC > 1.5) in CD patients and controls, and the levels of four differed in patients with active and inactive CD. PLS-DA revealed models with smallest error rate for controls in bacterial component and inactive disease in metabolites.

**Conclusion:**

A complex interrelationship may exist between gut dysbiosis, miRNA profiling and SCFA level in response to intestinal inflammation.

## Background

Microbial communities of the gastro-intestinal tract consist of at least 100 trillion microorganisms [1, 2]. This complex ecosystem trains the immune system, protects against opportunistic pathogens, harvests nutrients and energy from the diet, and ferments non-digestible carbohydrates [3]. A relatively stable composition of gut microbiota within individuals is maintained by diet, sanitation, antibiotics, aging, and other factors [4], commensal microbial compounds promote steady state hematopoiesis, shape-composition, activation status, immune cell repertoires, and vigilance of the innate and adaptive immune systems against different stimuli [5–7].

Infection of the alimentary tract with opportunistic pathogens usually leads to acute gastroenteritis, and disruption of the ecological organization of normal gut microbiota, called dysbiosis, may lead to immune system defects associated with various chronic human disorders, including the inflammatory bowel diseases (IBDs) - Crohn’s disease (CD) and ulcerative colitis (UC) [8, 9]. CD and UC are classic polygenic disorders, associated with almost 200 risk loci, including more than 30 loci specific for CD [10–12]. These genetic loci were associated with multiple intestinal immunopathological processes that occur in response to intestinal dysbiosis [10, 13].

Microbial dysbiosis in IBD is associated with a reduction in bacterial diversity, with colonization by pathogenic bacteria deranging the stability of the entire bacterial community [14]. CD dysbiosis is associated with reductions in *Bacteroidetes* and *Firmicutes*, increases in *Gammaproteobacteria* and *Enterobacteriaceae*, increases in the isolation of adherent-invasive *Escherichia coli* from ileal CD biopsies, and reductions in *Faecalibacterium prausnitzi* commensal, strains of protective bacteria with anti-inflammatory properties [15–20].

About 90% of the total microbial number in adult gut belong to the two most abundant phyla, *Bacteroidetes* and *Firmicutes*, which together with Actinobacteria and Verrucomicrobia are the main producers of short chain fatty acids (SCFAs) generated by anaerobic fermentation from dietary carbohydrates (i.e. fiber) and amino acids (i.e. L-glutamate, L-lysine) [21–23]. SCFAs are aliphatic saturated carboxylic acids with acetate, propionate, and butyrate being the most abundant SCFAs in colon and stool [24]. SCFAs are the primary energy source for colonocytes and maintain intestinal homeostasis through its anti-inflammatory activities. At the cellular level, SCFAs can influence the proliferation and differentiation of colonic regulatory T-cells (Treg) cells, as well as alter their gene expression [25–27]. The reduced SCFA levels in patients with IBD result from lower abundance of SCFA-producing bacteria, especially those of the phylum Firmicutes [28, 29], but also may relate to reduced fiber consumption. While a long-term intake of dietary fiber has been associated with a lower risk of CD development [30], and a high fiber diet is not harmful and seems to be favorable for CD [31], high fiber consumption is not practically advocated, particularly in the active disease [32].

Micro-RNAs (miRNAs) are small, non-coding particles that play a role in human physiology and pathology, with dysregulated miRNAs contributing to autophagy, intestinal inflammation, and fibrosis [33, 34]. miRNAs, secreted by intestinal epithelial cells, are detected in stool and play a role in crosstalk between microbiota and their hosts [35, 36]. miRNAs can infiltrate bacterial cells, regulate bacterial gene transcription, and promote bacterial growth [36]. miR-223 and miR-1246, which are generally present at high levels in stool, were associated with intestinal inflammation, including in patients with IBD [37], whereas fecal miRNAs may serve as biomarkers of IBD [38].

Although inferring interactions across omics datasets has multiple statistical challenges, the integration of multi-omics datasets pointed the role of microbially produced metabolites and IBD development [39, 40]. However, both IBD host and microbial features that may relate to the gut microbiome should be further characterized, particularly in direct association with the host epithelium and corresponding molecular changes [41].

The aim of this study was to analyze the potential relationship between gut dysbiosis, stool miRNA composition and SCFA level in response to CD intestinal inflammation.

## Methods

### Samples

The study cohort consisted of 15 CD patients, five women and ten men, of median age 32 years (range, 20–62 years), with adequate clinical information; and nine healthy control individuals, six women and three men, of median age 36 years (range, 26–41 years). CD was diagnosed by experienced gastroenterologists during a standard diagnostic work-up, using the Porto criteria modified in accordance with ECCO guidelines. Patients were recruited during a course of hospital treatment or during a scheduled visit to the out-patient department at the Department of Gastroenterology, Hepatology and Clinical Oncology, Medical Center for Postgraduate Education, Warsaw. Disease activity was determined by measuring the CD activity index (CDAI) [42], and the CD patients were assigned to two subgroups. According to the limit for a CDAI score of 220, seven patients in remission or with mild CD were considered to have inactive disease, and eight patients with moderate to severe CD were considered to have active disease. Most patients had ileocolic inflammation, and their stool samples were collected before medication was administered. Clinical characteristics of the enrolled patients is presented in Table 1. Control individuals, all of whom were hospital employees, reported themselves as being healthy. All enrolled patients and controls were Polish Caucasians.

**Table 1:** The clinical characteristics of the enrolled patients;1-Female, 2-Male

Sex	Age (years)	Inflammation	CD activity	Previous treatment		
				Immunosupressants	Glucocorticoids	Biological therapy
1	20–25	ileocolonic	active	yes	no	No
2	20–25	ileocolonic	active	no	yes	No
1	30–35	colonic	active	no	no	Yes
2	45–50	ileocolonic	active	no	yes	No
2	60–65	ileal	active	yes	no	No
2	50–55	colonic	active	no	no	Yes
2	40–45	ileocolonic	active	no	no	Yes
1	35–40	ileocolonic	active	yes	no	No
2	30–35	ileocolonic	inactive	yes	no	Yes
2	30–35	ileocolonic	inactive	yes	no	Yes
2	30–35	ileocolonic	inactive	yes	no	Yes
1	35–40	ileocolonic	inactive	no	no	Yes
1	20–25	ileocolonic	inactive	yes	no	Yes
2	25–30	colonic	inactive	no	no	Yes
2	20–25	ileocolonic	inactive	yes	no	Yes

The study was performed in accordance with the ethical standards of the local bioethical committee and in accordance with the principles of the 1964 Declaration of Helsinki.

### Stool collection and preparation

Subjects were provided a stool collection kit, consisting of a Styrofoam box, tubes, and spatulas for stool samples; an ice pack; and a disposable bag. A stool sample from a single bowel movement was collected and immediately frozen at − 20 °C. Aseptic techniques using a disposable scalpel were utilized to scrape off approximately 200, 200, and 100 mg of each stool sample for the extraction of DNA, miRNA, and SCFA, respectively.

### Fecal DNA extraction and 16S rRNA sequencing

DNA was extracted from stool samples using QIAamp Fast DNA Stool Mini Kits (Qiagen) according to the manufacturer’s directions, except that frozen stool samples, weighing approximately 180 mg, were mixed with 1 ml InhibitEx Buffer and incubated at 95 °C for 5 min to lyse Gram-positive bacteria. DNA concentrations were measured using a Nanodrop ND-1000 spectrophotometer.

16S rRNA was sequenced on an Ion Torrent Personal Genome Machine (PGM) platform using Ion PGM™ Hi-Q™ View OT2 and Ion PGM Hi-Q View Sequencing Kits. 16S rRNA libraries were prepared using Ion 16S Metagenomic Kits (which allows a consensus view across 6 regions V2, V3, V4, V6–7, V8 and V9), as previously described [43].

### Fecal miRNA extraction and Nanostring nCounter miRNA profiling

Fecal miRNA was isolated from stool samples (approximately 200 mg) using mirVana miRNA Isolation Kits, according to the manufacturer’s protocol. miRNA was screened using 100 ng miRNA, as recommended, and analyzed with nCounter human v2 miRNA expression assay kits, which allow detection of 800 human miRNAs. Hybridization was performed on the nCounter Prep Station, and miRNA was detected with an nCounter Digital Analyzer.

### Short chain fatty acids profiling

Metabolites were extracted and derivatized as described [44] with modifications. Briefly, frozen stool samples weighing approximately 100 mg were each placed in 2 ml tubes containing ceramic beads and 1 ml of 10% isobutanol. The samples were mechanically homogenized twice, for 2 min each, on an HT Lysing Homogenizer at 1500 rpm with a 30 s interval. The samples were centrifuged at 12000 x g for 6 min, and 675 μl of each supernatant was transferred to a new Eppendorf tube. After adding 125 μl of 20 mM NaOH and 400 μl chloroform to each sample, the samples were vortexed and centrifuged at 21000 x g for 2 min. A 400 μl aliquot of each upper aqueous phase was transferred to a new tube; 100 μl pyridine and 80 μl isobutanol was added; and the volume of each sample was adjusted to 650 μl by adding ultra-pure water.

The calibration standards formate, acetate, propionate, butyrate, isobutyrate, and valerate were obtained from Sigma-Aldrich (St. Louis, MO), at the desired concentrations were combined with 125 μl of 20 mM NaOH, 100 μl pyridine and 80 μl isobutanol, and the volume of each was adjusted to 650 μl with ultra-pure water.

Samples and calibration standards were derivatized by adding 50 μl chloroformate isobutyl to each 650 μl sample. The lid of each tube was opened for 1 min to release gases produced during the reaction. The samples were vortexed for 1 min, and 170 μl hexane was added to each, and the samples were again vortexed. After centrifuging the samples at 20000 x g for 5 min, a 170 μl aliquot of each upper Isobutyl-hexane phase was transferred to an autosampler vial.

SCFAs were quantified by GC/MS on an Agilent 7000D Triple Quadrupole mass spectrometer coupled to a 7890 GC System with a G4513A autosampler and a VF-5 ms column (30 m, 0.25 mm, 0.50 μm). The temperatures of the injector, ion source, quadrupole, and transfer line were set at 260 °C, 250 °C, 150 °C, and 310 °C, respectively. Helium carrier gas flow was maintained at 1 ml/min. A 1 μl aliquot of each derivatized sample was injected at a split ratio of 25:1, with the solvent delay set at 3 min. The initial column temperature of 40 °C was maintained for 5 min and then ramped at a rate of 10 °C/min to 310 °C, which was maintained for 10 min. MS data were collected in full scan mode from m/z 15–300 at a frequency of 4 scans per second. The target ion (m/z) of formate, acetate, propionate, isobutyrate, butyrate, and valerate were 56, 56, 57, 71, 71, and 85, respectively.

### Statistical analysis

#### 16S rRNA analysis

Unmapped BAM files were converted to FASTQ using Picard’s [45] SamToFastq. Additional steps of the analysis were performed using Mothur [46] version 1.38 software. FASTQ files were converted to the FASTA format. For analyses, only the sequences that were 200–300 bases in length, with an average base quality of 20 in a sliding window of 50 bases, and a maximum homopolymer length of 10 were kept. Chimeric sequences were identified with the UCHIME [47] algorithm using default parameters, with internal sequence collection as the reference database. Chimeric sequences were removed, and the remaining 16S rRNA sequences were classified using the Wang method and the SILVA [48] bacterial 16S rRNA database for reference (release 132), at an 80% bootstrap cut-off. The non-parametric Shannon diversity index and the Chao richness index were determined with Mothur. Differences in taxa prevalence were determined with Fisher’s exact test corrected with Benjamini-Hochberg procedure. Between group differences in the abundance of taxa were assessed with DESeq2 [49], using Wald’s test to determine the statistical significance of fold-change difference The default DESeq2 normalization (based on median of ratios) and dispersion estimations were applied. The normalization and dispersion estimations were counted for the whole dataset and appropriate comparisons were extracted with DESeq2 contrast option. The *p*-values were then adjusted for multiple testing with Benjamini-Hochberg procedure. Taxa with median normalized count value smaller than 5 in both groups or taxa with adjusted p-value in Fisher’s exact test for prevalence smaller than 0.05 were excluded from analysis. Differences in diversity indices values were assessed using the Kruskall-Wallis test, followed by the post-hoc Mann-Whitney U-test.

#### miRNA analysis

Data were processed and analyzed with nSolver™ Analysis Software 4.0. Levels of expression were normalized relative to the geometric mean of the 100 miRNAs with the highest numbers of counts. Due to counts of negative probes, all results were corrected using a background threshold value set at 40 counts. Significant differences were determined by *t*-tests. miRNA was considered differentially expressed when FC > 1.5 and corrected *p-*value < 0.05 (Benjamini-Hochberg algorithm).

#### SCFA analysis

Data were analyzed by MassHunter software. SCFA concentrations were obtained from calibration curve. SCFA values were log10 transformed. Significant differences were determined by ANOVA test.

#### Integrated analysis

Taxonomic, miRNA, and metabolite data (including Partial Least Squares Discriminant Analysis (PLS –DA) were integrated using the DIABLO function of the MixOmics [50] package. The numbers of components and variables were tuned according to the tutorial present at http://mixomics.org/mixdiablo/case-study-tcga/. The number of components was determined after M-fold cross-validation (with 5 folds and 100 repeats) performance assessment of full PLS model, using the number given by Weighted Prediction for error rate. The number of variables in each component was determined with mixOmics tune.block.splsda function with the same validation method as above and distances between centroids as distance measure. Final model performance was assessed with M-fold cross-validation with 5 folds and 1000 repeats. The correlation between variables relevant both in integrated models and statistically significant in previous tests was determined with Spearman’s coefficient. Multiple testing correction for corresponding *p*-values for coefficients was performed with FDR procedure.

## Results

### 16S rRNA microbiome survey

An average of 92,000 reads were generated (minimum – 57,808, maximum – 145,096), fulfilling the quality criteria, described in Materials and Methods. Of the 432 taxa identified in these samples, 81 were present at level higher than 0.1% of reads. The five most abundant bacterial families were *Bacteroidaceae, Burkholderiaceae, Ruminococcaceae, Lachnospiraceae*, and *Prevotellaceae*.

**Fig. 1:**
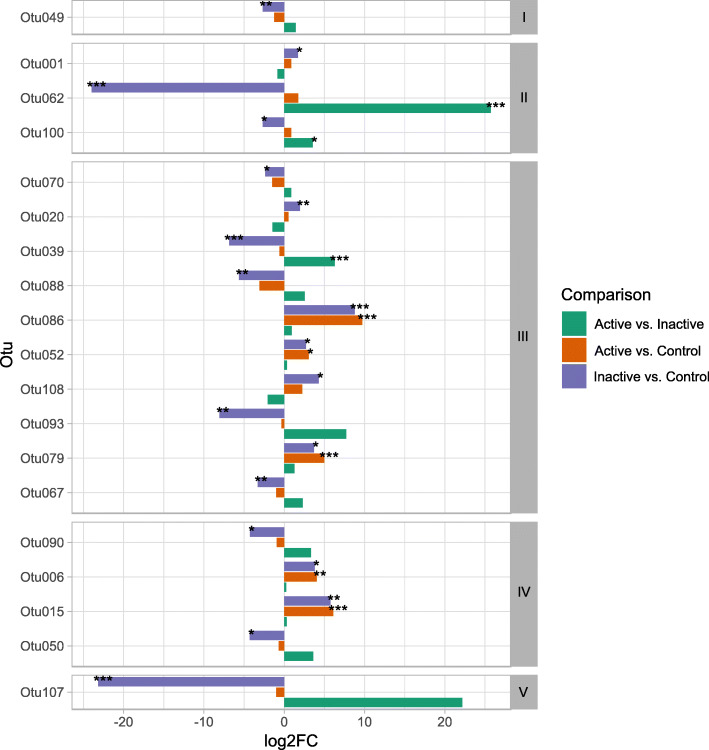
Statistically significant taxonomic changes occurring in at least one comparison of control subjects, patients with inactive CD, and patients with active CD; Phyla: I – Bacteria unclassified, II – Bacteroidetes, III – Firmicutes, IV – Proteobacteria, V – Verrucomicrobia; *P*-value: * < 0.05, ** < 0.01, *** < 0.001

In pairwise comparisons, 9 and 23 taxa abundances differentiated healthy controls and CD patients with active and inactive disease, respectively (Fig. 1). When controls were compared with patients with active disease, *Enterobacteriaceae*, including genus *Escherichia-Shigella* and *Lachnospiraceae,* including *Tyzerella_4* were over-represented. Similar bacteria were over-represented in patients with inactive CD. In addition, *Bacteroides* were over-represented, whereas *Pasteurellaceae* and the genus *Coprobacter* were under-represented, in patients with inactive CD*.* Overall, six taxa (including *Enterobacteriaceae*, *Escherichia-Shigella*, *Tyzzerella_4, Erysipelotrichaceae_genus, Erysipelatoclostridium*, and *Flavonifractor*) differed in healthy controls and both groups of CD patients (Table 2, Supplementary Tables S1 and S2).

**Table 2:** Taxa differing between healthy controls and patients with active and inactive CD

Otu	log2FC	padj	Taxonomy	Comparison
Otu086	9.73	8.05E-05	Bacteria;Firmicutes;Clostridia;Clostridiales;Lachnospiraceae;Tyzzerella_4;	CONTROL vs Active
Otu114	6.46	3.16E-04	Bacteria;Firmicutes;Erysipelotrichia;Erysipelotrichales;Erysipelotrichaceae;Erysipelotrichaceae_ge;	CONTROL vs Active
Otu015	6.09	5.21E-04	Bacteria;Proteobacteria;Gammaproteobacteria;Enterobacteriales;Enterobacteriaceae;Escherichia-Shigella;	CONTROL vs Active
Otu079	4.98	5.28E-04	Bacteria;Firmicutes;Erysipelotrichia;Erysipelotrichales;Erysipelotrichaceae;Erysipelatoclostridium;	CONTROL vs Active
Otu123	3.74	2.51E-03	Bacteria;Firmicutes;Clostridia;Clostridiales;Ruminococcaceae;Anaerotruncus;	CONTROL vs Active
Otu006	4.04	7.36E-03	Bacteria;Proteobacteria;Gammaproteobacteria;Enterobacteriales;Enterobacteriaceae;	CONTROL vs Active
Otu156	6.31	1.47E-02	Bacteria;Firmicutes;Clostridia;Clostridiales;Lachnospiraceae;Hungatella;	CONTROL vs Active
Otu052	3.06	1.85E-02	Bacteria;Firmicutes;Clostridia;Clostridiales;Ruminococcaceae;Flavonifractor;	CONTROL vs Active
Otu205	−3.69	1.85E-02	Bacteria;Firmicutes;Clostridia;Clostridiales;Lachnospiraceae;Lachnospiraceae_UCG-008;	CONTROL vs Active
Otu062	−23.98	2.91E-27	Bacteria;Bacteroidetes;Bacteroidia;Bacteroidales;Barnesiellaceae;Coprobacter;	CONTROL vs Inactive
Otu107	−23.16	1.80E-14	Bacteria;Verrucomicrobia;Verrucomicrobiae;Verrucomicrobiales;Akkermansiaceae;Akkermansia;	CONTROL vs Inactive
Otu039	−6.86	3.92E-05	Bacteria;Firmicutes;Clostridia;Clostridiales;Lachnospiraceae;Lachnospiraceae_NK4A136_group;	CONTROL vs Inactive
Otu086	8.79	4.34E-04	Bacteria;Firmicutes;Clostridia;Clostridiales;Lachnospiraceae;Tyzzerella_4;	CONTROL vs Inactive
Otu015	5.78	1.38E-03	Bacteria;Proteobacteria;Gammaproteobacteria;Enterobacteriales;Enterobacteriaceae;Escherichia-Shigella;	CONTROL vs Inactive
Otu020	1.97	1.38E-03	Bacteria;Firmicutes;Clostridia;Clostridiales;Lachnospiraceae;Blautia;	CONTROL vs Inactive
Otu114	5.79	1.38E-03	Bacteria;Firmicutes;Erysipelotrichia;Erysipelotrichales;Erysipelotrichaceae;Erysipelotrichaceae_ge;	CONTROL vs Inactive
Otu067	−3.29	2.20E-03	Bacteria;Firmicutes;Firmicutes_unclassified;Firmicutes_unclassified;Firmicutes_unclassified;	CONTROL vs Inactive
Otu120	−3.90	3.45E-03	Bacteria;Firmicutes;Clostridia;Clostridiales;Family_XIII;Family_XIII_AD3011_group;	CONTROL vs Inactive
Otu049	−2.68	5.36E-03	Bacteria;Bacteria_unclassified;Bacteria_unclassified;Bacteria_unclassified;Bacteria_unclassified;	CONTROL vs Inactive
Otu093	−8.06	5.36E-03	Bacteria;Firmicutes;Clostridia;Clostridiales;Ruminococcaceae;Ruminococcaceae_UCG-014;	CONTROL vs Inactive
Otu088	−5.64	6.05E-03	Bacteria;Firmicutes;Clostridia;Clostridiales;Lachnospiraceae;Lachnospiraceae_UCG-001;	CONTROL vs Inactive
Otu006	3.80	1.19E-02	Bacteria;Proteobacteria;Gammaproteobacteria;Enterobacteriales;Enterobacteriaceae;	CONTROL vs Inactive
Otu079	3.72	1.72E-02	Bacteria;Firmicutes;Erysipelotrichia;Erysipelotrichales;Erysipelotrichaceae;Erysipelatoclostridium;	CONTROL vs Inactive
Otu001	1.70	2.07E-02	Bacteria;Bacteroidetes;Bacteroidia;Bacteroidales;Bacteroidaceae;Bacteroides;	CONTROL vs Inactive
Otu090	−4.26	2.07E-02	Bacteria;Proteobacteria;Deltaproteobacteria;Desulfovibrionales;Desulfovibrionaceae;	CONTROL vs Inactive
Otu108	4.30	2.07E-02	Bacteria;Firmicutes;Clostridia;Clostridiales;Ruminococcaceae;Oscillospira;	CONTROL vs Inactive
Otu070	−2.36	2.87E-02	Bacteria;Firmicutes;Clostridia;Clostridiales;Clostridiales_unclassified;	CONTROL vs Inactive
Otu118	−2.97	2.87E-02	Bacteria;Firmicutes;Clostridia;Clostridiales;Ruminococcaceae;Ruminococcaceae_UCG-013;	CONTROL vs Inactive
Otu050	−4.30	3.28E-02	Bacteria;Proteobacteria;Gammaproteobacteria;Pasteurellales;Pasteurellaceae;	CONTROL vs Inactive
Otu052	2.70	3.62E-02	Bacteria;Firmicutes;Clostridia;Clostridiales;Ruminococcaceae;Flavonifractor;	CONTROL vs Inactive
Otu100	−2.69	4.95E-02	Bacteria;Bacteroidetes;Bacteroidia;Bacteroidia_unclassified;Bacteroidia_unclassified;	CONTROL vs Inactive
Otu163	4.87	4.95E-02	Bacteria;Firmicutes;Erysipelotrichia;Erysipelotrichales;Erysipelotrichaceae;Faecalitalea;	CONTROL vs Inactive

(*Abbreviations*: *Otu* Taxon number, *log2FC* base 2 logarithm of fold difference between groups, *padj* FDR-adjusted p-value derived from DESEQ2 results, *taxonomy* Taxonomic classification, *comparison* groups showing a difference in that taxon)

In addition, the levels of four taxa differed in patients with active and inactive CD, with *Coprobacter* showing the greatest difference (Supplementary Table S3). All differences are present after Benjamini – Hochberg procedure multiple testing correction.

**Fig. 2:**
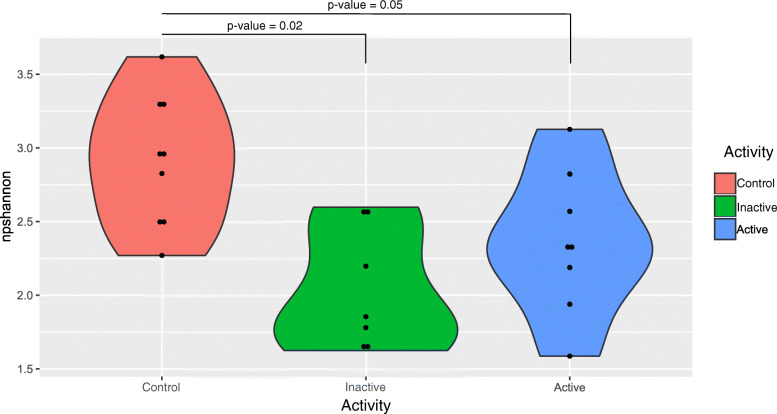
Differences in alpha diversity, as represented by Shannon index. *P*-values are given for the Mann–Whitney U-test and adjusted with FDR correction

Compared with controls, both groups of CD patients showed lower alpha diversity (Kruskall–Wallis test, *p*-value = 0.01 for Np Shannon index; Fig. 2). There were no significant differences, however, in species richness.

### miRNA profiling

Stool miRNA profiles in the CD subgroups and healthy controls were determined by Nanostring screening, which allowed detection of 800 human miRNA, with 149 miRNAs above the threshold, which had been set at 40 counts.

Before multiple testing correction, levels of 13 miRNAs significantly differed (FC > 1.5 and *p-*value < 0.05) CD patients from controls. Of these, four (hsa-miR-223-3p, hsa-miR-142-3p, hsa-miR-16-5p, and hsa-miR-23a-3p) were more abundant in patients with CD, and nine (hsa-miR-577, hsa-miR-379-5p, hsa-miR-642a-3p, hsa-miR-26b-5p, hsa-miR-361-5p, hsa-miR-194-5p, hsa-miR-202-3p, hsa-miR-155-5p, and hsa-miR-141-3p) were less abundant in CD patients (Table 3). After multiple test correction with the Benjamini – Hochberg procedure, four miRNAs (hsa-miR-577, hsa-miR-379-5p, hsa-miR-642a-3p, hsa-miR-26b-5p) remained differential; all of them were less abundant in patients with CD.

**Table 3:** miRNA levels differing in CD patients and healthy controls. Bold type indicates the most significantly different miRNAs

Statistics				Counts								Probe Name
				Control				CD				
Padjust	*P*-value	t-statistic	FC	Upper 95% CI	Lower 95% CI	SD	Control	Upper 95% CI	Lower 95% CI	SD	mean	
2.27E-02	1.52E-04	−5.21	−4.94	467.18	170.93	192.70	319.05	100.07	31.49	61.92	65.78	hsa-miR-577
3.10E-02	6.53E-04	−3.97	−3.65	958.18	427.83	344.98	693.00	457.24	98.43	323.96	277.83	hsa-miR-379-5p
3.10E-02	6.61E-04	−4.03	−4.18	993.98	403.93	383.81	698.95	530.38	73.58	412.44	301.98	hsa-miR-642a-3p
3.10E-02	8.33E-04	−3.98	−5.10	3688.71	1227.90	1600.70	2458.30	925.97	227.70	630.45	576.83	hsa-miR-26b-5p
1.48E-01	4.97E-03	3.22	7.68	121.66	15.04	69.36	68.35	11,532.45	N/A	12,490.41	4615.49	hsa-miR-223-3p
3.06E-01	1.23E-02	−2.75	−3.09	957.20	182.63	503.83	569.91	396.00	79.03	286.19	237.52	hsa-miR-361-5p
3.94E-01	2.25E-02	2.53	3.26	69.14	28.49	26.44	48.81	2920.51	N/A	3303.39	1091.15	hsa-miR-142-3p
3.94E-01	2.26E-02	−2.46	−2.17	508.60	202.53	199.09	355.57	301.91	91.82	189.68	196.87	hsa-miR-194-5p
3.94E-01	2.57E-02	−2.41	−2.37	387.17	165.77	144.02	276.47	237.50	66.22	154.65	151.86	hsa-miR-202-3p
3.94E-01	3.44E-02	2.34	2.39	40.00	40.00	0.00	40.00	915.66	N/A	936.19	397.21	hsa-miR-16-5p
3.94E-01	3.79E-02	−2.22	−2.74	6467.88	1662.47	3125.80	4065.17	3449.42	1007.05	2205.17	2228.23	hsa-miR-155-5p
3.94E-01	3.85E-02	2.25	2.21	63.15	30.85	21.01	47.00	536.93	N/A	487.63	266.89	hsa-miR-23a-3p
3.94E-01	4.73E-02	−2.23	−1.54	107.06	48.42	38.14	77.74	58.32	35.97	20.18	47.14	hsa-miR-141-3p

In addition, the levels of 12 miRNAs differed significantly in stool samples from patients with active disease and healthy controls, with five being more abundant and seven being less abundant in patients with active CD. After multiple testing correction, three of them (hsa-miR-379-5p, hsa-miR-577, hsa-miR-26b-5p), all less abundant in patients with CD, remained differential (Table 4).

**Table 4:** miRNA levels differing in patients with active CD and healthy controls. Bold type indicates the most significantly differed miRNAs

Statistics				Counts								Probe Name
				Control				Active CD				
Padjust	*P*-value	t-statistic	FC	Upper 95% CI	Lower 95% CI	SD	mean	Upper 95% CI	Lower 95% CI	SD	Active	
5.33E-03	8.51E-05	−5.40	−4.59	958.18	427.83	344.98	693.00	212.23	91.98	71.92	152.11	hsa-miR-379-5p
5.33E-03	9.26E-05	−6.34	−5.91	467.18	170.93	192.70	319.05	57.79	32.79	14.95	45.29	hsa-miR-577
2.06E-02	5.38E-04	−4.40	−5.83	3688.71	1227.90	1600.70	2458.30	638.45	156.17	288.44	397.31	hsa-miR-26b-5p
7.68E-02	2.67E-03	−3.98	−4.70	993.98	403.93	383.81	698.95	370.73	34.95	200.82	202.84	hsa-miR-642a-3p
1.79E-01	7.79E-03	3.07	3.11	19,580.00	N/A	13,149.21	9472.62	39,519.23	10,096.10	17,597.13	24,807.66	hsa-miR-1246
2.73E-01	1.47E-02	3.09	11.65	121.66	15.04	69.36	68.35	5015.90	N/A	3131.06	2398.27	hsa-miR-223-3p
2.73E-01	1.66E-02	−2.70	−2.63	443.50	166.13	180.42	304.81	168.83	53.07	69.23	110.95	hsa-miR-944
3.43E-01	2.39E-02	2.79	4.17	69.14	28.49	26.44	48.81	709.34	49.38	394.70	379.36	hsa-miR-142-3p
3.52E-01	3.15E-02	−2.40	−3.15	957.20	182.63	503.83	569.91	431.54	1.03	257.47	216.28	hsa-miR-361-5p
3.52E-01	3.35E-02	2.34	2.49	351.20	77.52	178.02	214.36	760.10	189.66	341.16	474.88	hsa-miR-4516
3.52E-01	3.36E-02	2.57	3.00	63.15	30.85	21.01	47.00	496.06	N/A	301.27	244.19	hsa-miR-23a-3p
4.34E-01	4.92E-02	−2.32	−1.62	119.32	36.74	53.72	78.03	40.00	40.00	0.00	40.00	hsa-miR-378b

Moreover, the levels of seven miRNAs differed significantly in stool samples from patients with inactive disease and healthy controls, with all seven being more abundant in patients with inactive CD (Table 5). The levels of two miRNA (hsa-miR-1246 and hsa-miR-4488) differed significantly in patients with active and inactive CD (Table 6). In both cases they turned out statistically insignificant after multiple test correction.

**Table 5:** miRNA levels differing in patients with inactive CD and healthy controls. Bold type indicates the most significantly differed miRNAs

Statistics				Counts								Probe Name
				Control				Inactive CD				
Padjust	*P*-value	t-statistic	FC	Upper 95% CI	Lower 95% CI	SD	mean	Upper 95% CI	Lower 95% CI	SD	mean	
4.34E-01	4.51E-03	3.43	4.03	467.18	170.93	192.70	319.05	169.21	9.18	86.52	89.20	hsa-miR-577
4.34E-01	1.04E-02	3.01	2.81	508.60	202.53	199.09	355.57	188.60	65.57	66.51	127.08	hsa-miR-194-5p
4.34E-01	1.57E-02	3.06	1.72	107.06	48.42	38.14	77.74	40.00	40.00	0.00	40.00	hsa-miR-141-3p
4.34E-01	1.73E-02	2.99	1.87	128.28	49.24	51.41	88.76	40.00	40.00	0.00	40.00	hsa-miR-192-5p
4.34E-01	1.91E-02	2.91	1.74	113.97	48.41	42.65	81.19	43.05	38.72	2.34	40.89	hsa-miR-200b-3p
4.34E-01	3.44E-02	2.45	4.38	3688.71	1227.90	1600.70	2458.30	1576.66	N/A	859.24	782.00	hsa-miR-26b-5p
4.34E-01	3.95E-02	2.32	1.72	129.33	51.57	50.58	90.45	60.93	31.55	15.89	46.24	hsa-miR-200a-3p

**Table 6:** miRNA levels differing in patients with active and inactive CD

Statistics				Counts								Probe Name
				Inactive CD				Active CD				
Padjust	*P*-value	t-statistic	FC	Upper 95% CI	Lower 95% CI	SD	mean	Upper 95% CI	Lower 95% CI	SD	mean	
6.42E-01	1.39E-02	2.92	3.81	11,753.33	2470.75	5018.45	7112.04	39,519.23	10,096.10	5018.45	24,807.66	hsa-miR-1246
6.42E-01	2.25E-02	2.69	2.55	78.96	31.05	25.90	55.00	284.48	66.52	25.90	175.50	hsa-miR-4488

### SCFA profiling

**Fig. 3:**
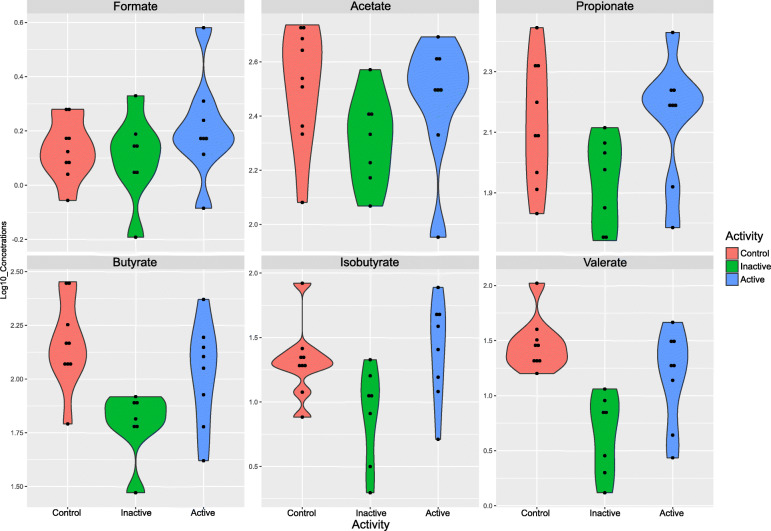
SCFA concentrations differing significantly in patients with active CD, inactive CD, and healthy controls

To test for significant association between CD status and SCFAs, the levels of six SCFAs (formate, acetate, propionate, isobutyrate, butyrate and valerate) were measured in stool extracts of patients with active CD, inactive CD, and controls. ANOVA showed significant differences in SCFA levels among these three groups (Fig. 3 and Supplementary Table S4).

Of the six SCFAs assayed, two, butyrate and valerate, differed significantly (*p*-value < 0.05, FC > 1.5) in CD patients and healthy controls. Although none of these SCFAs differed between patients with active disease and controls, three differed significantly in patients with inactive disease and controls, and four differed significantly in patients with active and inactive CD (Table 7).

**Table 7:** SCFA levels differing significantly in patients with active CD, inactive CD, and healthy controls

SCFA		Formate	Acetate	Propionate	Butyrate	Isobutyrate	Valerate
CD patients vs. Control	*p*-value	6.79E-01	2.01E-01	3.59E-01	**1.45E-02**	4.00E-01	**4.78E-03**
	FC	0.89	1.31	1.20	**1.71**	1.12	**2.51**
Active CD vs. Control	*p*-value	3.06E-01	6.59E-01	8.57E-01	2.15E-01	5.91E-01	1.03E-01
	FC	0.79	1.12	0.97	1.34	0.76	1.66
Inactive CD vs. Control	*p*-value	6.67E-01	6.61E-02	5.47E-02	**1.29E-03**	**2.54E-02**	**9.67E-05**
	FC	1.04	1.64	1.65	**2.48**	**2.46**	**5.96**
Active CD vs. Inactive CD	*p*-value	2.59E-01	1.82E-01	**3.90E-02**	**4.70E-02**	**2.55E-02**	**2.55E-02**
	FC	0.75	0.68	**0.59**	**0.54**	**0.31**	**0.28**
Control	mean	0.13	2.51	2.13	2.16	1.31	1.47
	median	0.12	2.54	2.09	2.16	1.30	1.44
	StDev	0.11	0.22	0.21	0.20	0.28	0.24
Inactive	mean	0.10	2.31	1.94	1.79	0.90	0.65
	median	0.14	2.33	1.98	1.81	1.04	0.84
	StDev	0.16	0.17	0.15	0.15	0.38	0.36
Active	mean	0.21	2.46	2.15	2.02	1.40	1.18
	median	0.18	2.51	2.20	2.08	1.50	1.27
	StDev	0.19	0.23	0.20	0.24	0.39	0.43

### Integrated analysis

**Fig. 4:**
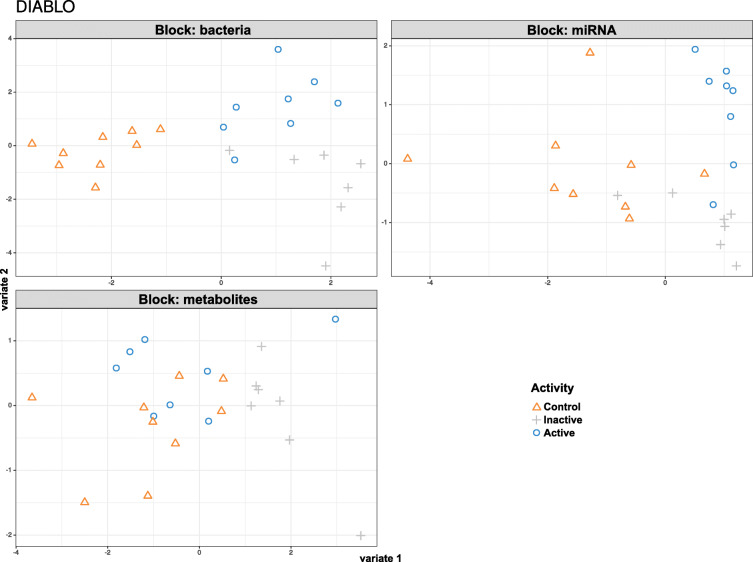
Results of PLSDA analysis, showing the first and second components

Partial Least Squares Discriminant Analysis (PLS-DA) (Fig. 4) identified models with smallest error rate for controls in bacteria (16% in the first component) and for inactive disease in metabolites (2% in the first component). Active CD cases showed a high level of error for all surveys, reaching the lowest level in the 16S rRNA sequencing survey. Model tuning resulted in a multi-omics biomarker panel with two components, including 15 bacterial taxa, five miRNAs, and five metabolites in both components. Of the 25 variables in the first component, 16 were influential in differentiating healthy controls from other outcomes, whereas the variables included in the second component were able to differentiate between patients with active and inactive CD (Supplementary Figure F1 and Supplementary Table S5).

Eleven correlations with high (> 0.6) values of Spearman’s coefficient were observed between variables that were either a part of final PLS-DA model or differentiated healthy controls from patients in the previous comparisons. However, none of them reached statistical significance after multiple testing correction (Supplementary Table S6).

## Discussion

Gut dysbiosis may be involved in the development of inflammatory disorders, including IBDs, type 1 diabetes, allergy, asthma, rheumatoid arthritis, and neurological diseases [51, 52]. In animal models of intestinal inflammation, dysbiosis initiated by acute pathogenic infection was associated with an impact on gut immune system that promoted chronic gut inflammation [53–55]. Despite UC and CD sharing many epidemiologic, immunologic, therapeutic, and clinical features, assessments of the microbiomes of patients with the respective diseases showed that they are two distinct subtypes of IBDs [56]. Although dysbiosis may play a major role in the pathogenesis of CD, it likely plays a much lesser role in the pathogenesis of UC [57, 58].

This study assessed the profiles of fecal microbiomes, SCFAs, and miRNAs in CD patients with active and inactive disease and in healthy controls. On the microbial level, we confirmed the occurrence of dysbiosis in CD patients, with these patients having lower α-diversity than healthy individuals, a finding in agreement with previous results [56]. Patients with inactive CD presented lower α-diversity than patients with active CD. This may be a result of a bacterial overgrowth, common in CD [59]. The abudance of *Bacteroidetes* were higher, whereas the amounts of *Firmicutes* were lower, in stool samples from CD patients than from controls. While *Bacteroidetes* are usually commensal species, they can induce the IBD in mouse models [60]; they also benefit hosts by being the main producers of butyrate [61]. A comprehensive overview of functional dysbiosis in the gut microbiome during IBD activity showed increases in facultative anaerobes, such as *E. coli which* correlates with inflammation status [17, 62], and decreases in obligate anaerobes, such as *Faecalibacterium prausnitzii* and *Roseburia hominis* [41]. Adult CD patients naïve to active treatment showed reductions in *Firmicutes* and *Clostridia*, and increases in *Bacteroidetes* [63]. A combination of 50 fecal bacterial taxa was recently shown to distinguish between active CD and CD in remission, with an AUC of 0.82, and the discriminatory power of the model was not influenced by disease locations and medications [64]. In sum, the bacterial community may reflect the CD status [58]. However, we could not relate the results to the patient’s bowel movement due to the lack of Bristol stool scale data.

The results of the present study confirmed decreases in the abundance of *Firmicutes* and *Bacteroidetes,* increases in *Enterobacteriaceae*, *Pasteurellaceae*, and *Veillonellaceae* and the presence of *Fusobacterium* in stool samples of CD patients [65, 66]. The distinguishing taxa, including *Lachnospiraceae*, *Ruminococcus*, *Roseburia*, *Blautia*, *F. prausnitzii* and *B. fragilis*, had been previously found as associating with disease activity in CD patients [67–71]. ,Although several studies showed that *F. prausnitzii* was decreased in the feces and intestinal tissues in patients with active CD [19, 67, 72], other studies found that *F. prausnitzii* was associated with remission [64]; however these findings were not confirmed in our study. An abundance of *B. fragilis* could distinguish between patients in remission and those with active disease [73, 74].

Gut microbiota, which live in a nutrient-rich environment, are involved in nutrient processing and maintaining energy homeostasis of the host [27, 57]. These bacteria also modulate the development of gut-associated lymphoid tissue and the colonization of the gut wall by intraepithelial lymphocytes, neutrophils, dendritic cells, ILC3s, mucosal-associated invariant T-cells, TCR αβ Th17 cells, TCR γδ IL-17-producing cells, Tregs, and immunoglobulin (Ig) A secreting plasma cells [57]. Microbiota also protect the host from opportunistic pathogens [75, 76] by producing bacteriocins and SCFAs. SCFAs show crosstalk with the intestinal barrier by stimulating mucus production by epithelial cells and the rearrangement of tight junction proteins, and with the systemic immune system after translocation from the gut to the bloodstream [77–79]. Differences between individuals with and without IBDs were most apparent in the metabolome showing a lower diversity of metabolite pools in IBD patients, a lower diversity that may be caused by poor nutrient absorption, greater water or blood content in the bowels, and shorter bowel transit times in individuals with active IBD [41]). IBD gut dysbiosis reduces the levels of SCFAs and secondary bile acids, while enhancing the levels of primary bile acids [41].

The best-studied microbial metabolites that influence immune system homeostasis are acetate, butyrate, and propionate [57]. Our SCFA analysis in stool samples showed that the levels of two out of the six SCFAs (butyrate, valerate) were significantly different in between CD patients and controls. Interestingly, four SCFA concentrations were significantly different between CD patients with inactive and active disease.

Butyrate can act as an energy source for normal colon epithelial cells, promoting their proliferation, but can also inhibit proliferation and induce apoptosis [80, 81]. Butyrate-producing bacteria are depleted in IBD patients, and probiotic treatment with these bacteria has therapeutic potential; supplementation with *F. prausnitzii* and a mix of six butyrate-producers in CD patients increased the butyrate production and reduced acetate levels, and the treatment with *B. pullicaecorum* 25-3 T and a mixture of six butyrate-producers improved epithelial barrier integrity in vitro [82].

Although modulation of microbiota in IBDs by probiotic butyrate-producing bacteria had little success in controlling the disease [82], transfer of fecal microbiota (FMT) from healthy donors to IBD patients induced a clinical response in 61% of patients with CD and only 22% of patients with UC [83, 84]. A higher proportion of *Lachnospiraceae* in donor stool was associated with a higher success rate of FMT [85], and recipients that responded to FMT exhibited increases in butyrate-producing *Lachnospiraceae* and *Ruminococcaceae* [86–89].

RNA interference by a single miRNA can regulate multiple genes, whereas a single gene may be targeted by many miRNAs [36]. Three miRNAs (mir-144, mir-519, and mir-211) were reported to affect the mucosa in CD [63]. The expression of miR-21 was found to be higher in inflamed colon mucosa of patients with active UC than in controls and UC patients in remission [90], whereas the levels of miR-21 and miR-155 were higher in colon mucosa of UC patients than in controls [91], with these two being among the most frequently and consistently deregulated miRNAs in IBD patients [38, 92–94].

Recently published study described 9 miRNAs (miR-15a-5p, miR-16-5p, miR-128-3p, miR-142-5p, miR-24-3p, miR-27a-3p, miR-223-3p, miR-223-5p, miR-3074-5p) and 8 miRNAs (miR-10a-5p, miR-10b-5p, miR-141-3p, miR-192-5p, miR-200a-3p, miR-375, miR-378a-3p, let-7 g-5p) which were significantly increased and decreased, respectively, in stool from CD patients. MiR-192-5p, miR-375, and miR-141-3p correlated with both the clinical CD activity index or CD endoscopic index of severity. The identified fecal miRNA alteration reflected pathophysiological mechanisms in CD, such as Th1 and Th17 inflammation, autophagy, and fibrotic processes [95].

Strong correlations were reported between miR-194-5p and let-7c-5p and certain bacterial families, such as *Enterobacteriaceae* [96]. These findings suggest that the intestinal microbiota may alter the profile of fecal miRNAs, which can mediate host-microbiota interactions and regulate intestinal health [36, 96, 97].

Our study identified 13 miRNAs that differed in CD patients and healthy controls. miRNA profiles were distinct in samples from patients with active (12 miRNAs) and inactive (seven miRNAs) CD. After applying multiple test correction, the numbers of miRNAs which remained differential between CD patients and healthy controls and between patients with active CD and control were reduced to 4 and 3, respectively. However, although multiple comparisons is typically demanded to minimize false positive results, this approach may also exclude true positive results. Therefore, we decided to present both corrected and uncorrected results, especially when our uncorrected data showed miR-155, miR-223-3p and miR-16-5p which were already reported as altered in IBD patient stool [37, 38]. MiR-223-3p is a pro-inflammatory miRNA, one of the critical components of IL23 inflammatory cascade. It targets claudin-8 which belongs to protein family responsible for the intestinal barrier homeostasis [98]. miR-16-5p can negatively regulate expression of adenosine receptor A2A and influence the NF-κΒ pathway. In turn, dysregulated NF-κΒ pathway is one of the key elements for CD development and progression [99]. .Furthermore, multiple test correction rejected two upregulated miRNA (miR-1246 and miR-223), reported previously as associating with intestinal inflammation [37, 100]. Of these, miR-1246 may be involved in NFAt proteins activation, which together with the activated NF-κΒ pathway may result in the expression of pro-inflammatory cytokines. MiR-577 and miR-26b-5p, both selected by our uncorrected testing, were found by others as downregulated in active CD and upregulated in inactive CD, and both were associated with colorectal cancer oncogenesis [101–103].

The correlations between fecal miRNAs and disease activity suggest that the miRNAs may be potential IBD biomarkers [38]. In addition, fecal miRNA levels were found to be associated with microbial composition in a manner that permits the miRNA profile, but not necessarily the microbiota, to indicate the inflammatory potential of the microbiota and its potential to contribute to inflammatory diseases, such as IBD [96]. Conversely, fecal miRNA-mediated inter-species gene regulation may facilitate host control of the gut microbiota [36].

Although microbial abundance may predict metabolite abundance profiles, integrative multi-omics approaches are challenging. Recently, the relationship between microbiota and metabolic changes was investigated using datasets generated under the integrative Human Microbiome Project and mmvec neural network architecture [40]. This study confirmed the core findings of previous investigations [41], including the co-occurrence of *Roseburia hominis* and multiple carnitines, as well as found a high correlation between *Klebsiella spp.* and IBD status, and between *Klebsiella* and several bile acids.

To assess interactions between microbe–metabolite-miRNA abundance, we used another multi-omics integrative method, which included PLSDA computation using the MixOmics [50] package. This method allowed investigation of common information across 16S sequencing, miRNA transcriptomic, and SCFA data. The PLSDA-based models using 10 miRNAs, 30 bacterial taxa, and six SCFAs had fairly low error rates for control group discrimination in bacterial component (16%) and for inactive CD in metabolite component (4%). Disappointingly, patients with active disease showed a high level of error for all variables tested. No statistically significant correlations were also uncovered, although 11 pairs presented with high correlation coefficient (absolute value above 0.6). Both of these issues may stem from relatively small sample size which served to generate a large volume of data.

## Conclusions

In summary, we created multi-omics profiles, characterizing the clinical status of CD patients. Bacterial taxa were the main contributors to the model, with 30 taxa present in the first two principal components, while metabolites achieved low error rates for patients with inactive disease. The main limitations of our study are a relatively small number of patients and, therefore, this preliminary research does not entitle us to draw final conclusions. However, we can speculate that the correlations among the three studied constituents suggest a complex mechanism underlying intestinal immunopathological processes.

## Supplementary information

**Additional file 1: Supplementary Table S1**. Taxa differentiating patients with active Crohn’s disease from healthy controls. sd - standard deviation, zeroes - number of zeroes in the group, IQR - interquartile range, log2FoldChange - base 2 logarithm from fold-change, stat - Wald’s test statistic, pvalue - unadjusted *p*-value, padj - FDR adjusted p-value for most abundant taxa, FC - fold-change, Taxonomy - taxonomic assignment, FisherTest - *p*-value in Fisher’s exact test for taxa prevalence, adjustFisher- FDR adjusted *p*-value for Fihser’s exact test. **Supplementary Table S2**. Taxa differentiating patients with inactive Crohn’s disease from healthy controls. sd - standard deviation, zeroes - number of zeroes in the group, IQR - interquartile range, log2FoldChange - base 2 logarithm from fold-change, stat - Wald’s test statistic, pvalue - unadjusted *p*-value, padj - FDR adjusted p-value for most abundant taxa, FC - fold-change, Taxonomy - taxonomic assignment, FisherTest - p-value in Fisher’s exact test for taxa prevalence, adjustFisher- FDR adjusted p-value for Fihser’s exact test. **Supplementary Table S3**. Taxa differentiating patients with inactive Crohn’s disease from patients with active disease. sd - standard deviation, zeroes - number of zeroes in the group, IQR - interquartile range, log2FoldChange - base 2 logarithm from fold-change, stat - Wald’s test statistic, pvalue - unadjusted p-value, padj - FDR adjusted p-value for most abundant taxa, FC - fold-change, Taxonomy - taxonomic assignment, FisherTest - p-value in Fisher’s exact test for taxa prevalence, adjustFisher- FDR adjusted p-value for Fihser’s exact test. **Supplementary Table S4**. SCFA log10 conetrations [ppm] for patients with active, inactive CD and control group. **Supplementary Table S4**. Error rates for PLS-DA models after M fold model vaildation. **Supplementary Table S6**. Correlation coefficients’ values for the relevant variables in the whole dataset, rho - Spearman’s correlation coefficient, pvalue - pvalue for the coefficient, padjusted - FDR adjusted pvalue, Taxonomy - taxonomic assignment (if applicable). **Supplementary Table SA**. GC/MS data.

**Additional file 2:.**

## Data Availability

The Metagenomics dataset supporting the conclusions of this article is available in the BioProject repository, https://www.ncbi.nlm.nih.gov/bioproject/603658 The Nanostring dataset supporting the conclusions of this article is available in the GEO repository, https://www.ncbi.nlm.nih.gov/geo/query/acc.cgi?acc=GSE144535 Mass Spectrometry data showed in supplementary Table SA.

## Abbreviations

SCFA: Short chain fatty acids
miRNA: Micro RNA
CD: Crohn’s Disease
IBD: Inflammatory Bowel Disease
CDAI: Crohn Disease Activity Index
GC/MS: Gas Chromatography – Mass Spectrometry
PLS-DA: Partial Least Squares Discriminant Analysis
StDev: Standard deviation
