# Supplementary figures and images for: In search for interplay between stool microRNAs, microbiota and short chain fatty acids in Crohn’s disease - a preliminary study
Source: BMC Gastroenterol. 2020 Sep 21;20:307. doi: 10.1186/s12876-020-01444-3 (PMC7507689; doi:10.1186/s12876-020-01444-3)

## Component 1

### OTU

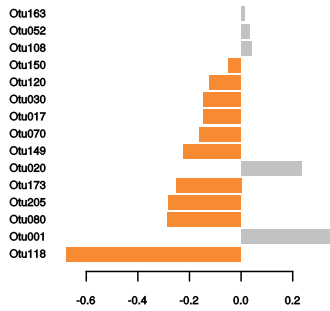

## Component 2

### OTU

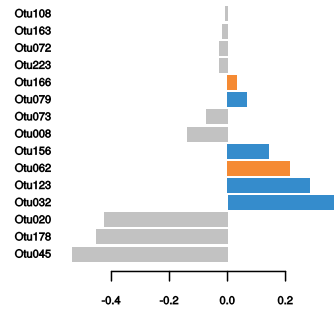

### miRNA

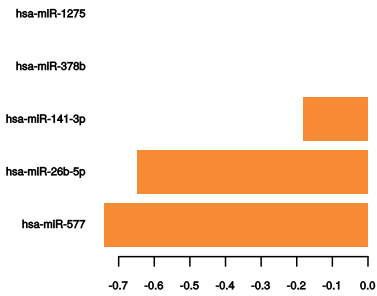

### miRNA

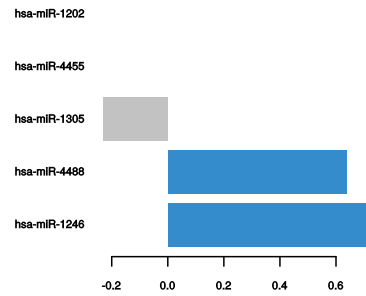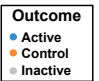

### metabolites

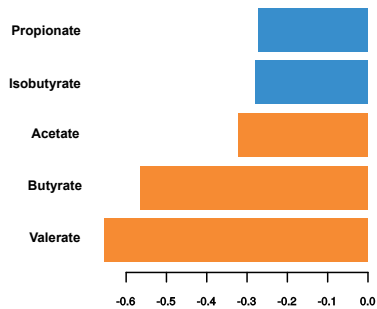

### metabolites

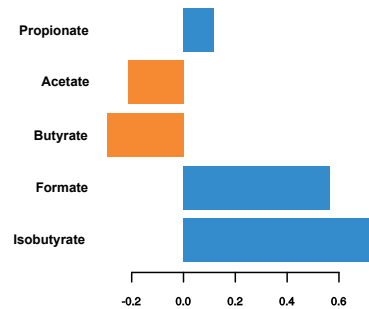

Supplement: Supplementary file 2 — Additional file 2:. [file 12876_2020_1444_MOESM2_ESM.pdf]
